# Financial Toxicity

**DOI:** 10.31557/APJCP.2020.21.2.289

**Published:** 2020

**Authors:** Luciana de Alcantara Nogueira, Bruna Eloise Lenhani, Dabna Hellen Tomim, Luciana Puchalski Kalinke

**Affiliations:** 1 *Department of Nursing, Federal University of Paraná,*; 2 *Erasto Gaertner Hospital, Curitiba, Brazil. *

**Keywords:** Neoplasms, financial toxicity, quality of life

## Abstract

**Objective:**

to identify evidence in the literature about financial toxicity in cancer patients during chemotherapy treatment.

**Methods:**

this is a mini review with search in the following databases: Cumulative Index to Nursing and Allied Health Literature, Latin American and Caribbean Health Sciences Literature, National Library of Medicine and Scopus. Articles were surveyed from July 2018 to February 2019 using the following descriptors and Boolean operators: “financial toxicity” OR “financial difficulties” AND “cancer” AND “chemotherapy”.

**Results:**

the search totaled 64 articles. After exclusion of duplicates that did not meet the inclusion criteria, the final sample was composed of five articles published in 2013, 2015, 2017 and 2018. Financial difficulty caused by cancer treatment and its impact on quality of life was a frequent expression used in the studies.

**Conclusion:**

studies show that financial toxicity damages the quality of life of patients. A scarcity of studies on the subject was also observed, possibly because this is a new concept.

## Introduction

The diagnosis of cancer is marked by physical, psychological, social, financial and other changes brought about by its treatment, the stage of the disease, and the stigma of death (Farinhas et al., 2013). These changes have an impact on activities of daily living and increase expenses with the health and quality of life (QOL) of patients and families.

The costs of cancer treatment are high, mainly due to the constant investment in research to find new therapies and equipment. The review by Connor et al., (2016) aimed at investigating financial toxicity in cancer treatment showed that the estimated annual expenditure with treatment exceeded US$ 200 billion in the United States in 2005, and represented 20% of total healthcare expenses. The review emphasized that even for patients with private health insurance, treatment costs can become a significant financial burden, leading to delayed onset of treatment and access to new therapies, and even personal bankruptcy (Meropol and Schulman, 2007; American Cancer Society, 2006).

In Brazil, cancer treatment may be covered by both private health insurance and the Unified Health System (SUS). The latter was created by Law nº 8,080 of September 19^th^, 1990, with the aim of ensuring comprehensive, universal and free access to health care for the entire population of the country. However, although patients have access to treatment, the disease affects other social spheres because it requires an eventual reduction of workload in activities and work leave due to side effects and adverse reactions of therapies, thus compromising the family budget.

A study by Nobrega and Lima (2014) conducted at a Chemotherapy Outpatient Clinic of a private hospital in the city of São Paulo - Brazil (BR) to identify the direct cost of procedures related to outpatient chemotherapy treatment among women with breast cancer found that the average cost per chemotherapy session was of approximately US $ 461, of which 93.75% were spent with drugs, 4.21% with materials, 1.60% with personnel, and 0.44% with solutions. However, these figures do not include the costs absorbed by patient, such as those with escort to therapy services, food/diet, adjuvant medications to minimize side effects at home, among others. Such out-of-pocket expenses significantly impact the family budget.

In the context of treatment-related costs absorbed by cancer patients, a new modality of adverse event in oncology is the so-called financial toxicity. This toxicity may impact on the physical, social and emotional function and cause a worsening in the QOL of patients and their families.

Financial toxicity is marked by the financial impact of cancer treatment on a patient’s life (Zafar et al., 2013). The clinical relevance of financial distress stands out as equivalent to physical and psychological distress. In fact, financial distress can affect multiple facets of life and ultimately the QOL (Delgado-Guay et al., 2015). It is found in the literature as a synonym for anxiety, overload, stress or financial burden of cancer treatment.

Financial toxicity is a wide-ranging term that encompasses the costs that patients have after the diagnosis of cancer, which impact on personal and family budget. Activities of daily living change due to the need for a differentiated diet, escorting, work absenteeism and expenses with caregivers among other demands. The treatment, whether covered by social security or not, generates costs with tests and/or adjunctive medications, which can financially burden patients and families.

Thus, the purpose of this mini review was to identify in the literature articles on financial toxicity in cancer patients during chemotherapy treatment.

## Materials and Methods

To guide the development of this mini review, the acronym PCC was used, standing for P - population: cancer patients, C - concept: financial toxicity, and C – context: chemotherapy.

The searches were conducted from July 2018 to February 2019 in four databases, namely, the Cumulative Index to Nursing and Allied Health Literature (CINAHL), Latin American and Caribbean Health Sciences Literature (LILACS), National Library of Medicine (PubMed), and Scopus. The following descriptors and Boolean operators were used: “financial toxicity” OR “financial difficulties” AND “cancer” AND “chemotherapy”.

Inclusion criteria were: language (Portuguese, Spanish, English); open access texts; no limitation as to year of publication. Exclusion criteria were: free communications, editorials, letters to the editor, updates, and review articles.

Studies were selected by two researchers independently, with contribution of a third researcher for reaching consensus when there was disagreement.

In the first stage, titles and abstracts were analyzed to verify if they addressed the theme and met the inclusion and exclusion criteria. In the second stage, the articles were read in full length.

Data on identification (type of journal, year of publication, authors, country), method, and main results of the study were extracted from the selected articles. A descriptive analysis of data was made and the articles were identified with the prefixes A1 to A5. 

## Results

**Figure 1:**
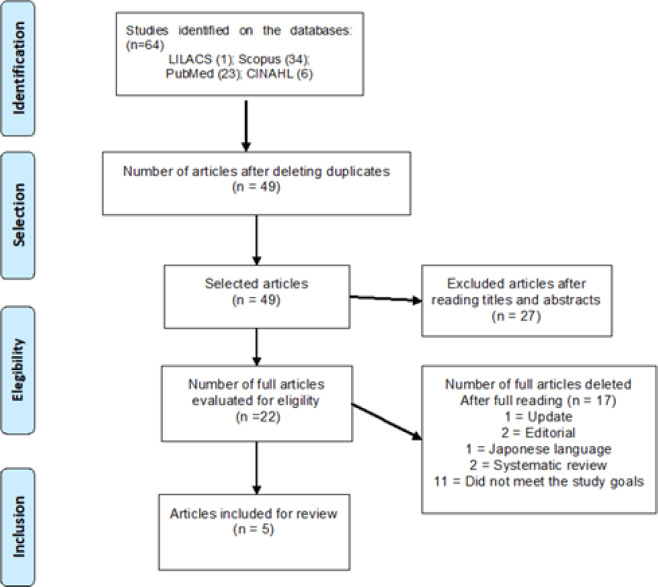
Flowchart of Search and Studies Selection

The initial search totaled 64 articles. After excluding duplicate articles that did not meet the inclusion criteria, the final sample was composed of five articles. Figure 1 illustrates the selection process according to the Preferred Reporting Items for Systematic Reviews and Meta- Analyses (PRISMA) methodology

The five selected articles (Table 1) were published in the years 2013, 2015, 2017 and 2018. Three articles were published in the USA, one in Norway and one in the United Kingdom. The studies had distinct methodological approaches: one was a prospective study, one was a descriptive/cross-sectional study, two were methodological studies, and one was an exploratory and observational study.

In A3, we identified the use of the European Organization for Research and Treatment of Cancer Quality of Life Core Questionnaire 30 (EORTC QLQ-C30) to assess the QOL of cancer patients. The tool brings a question that assesses whether the physical condition or medical treatment caused financial problems.

Financial difficulty caused by cancer treatment was a frequent expression used in the studies (A1 and A2) and was associated to noncompliance/discontinuity of care (A4). Evaluating the Comprehensive Score for financial Toxicity (COST) was the objective of two of the selected studies (A3 and A5). It is noteworthy that the articles related financial toxicity with quality of life.

**Table 1:** Presentation of the Research Results

	Author / year/country	Method	Objective	Sample	Main results
A2	Egestad H, Nieder C. 2015. Norway	Prospective Study	To analyze the deterioration of health-related quality of life in patients with head and neck cancer due to the financial issues associated with treatment.	67 patients with head and neck cancer	89% of patients provided treatment data on financial difficulties; there was a tendency to financial difficulty in the case of men under 65 years old; the highest score of financial difficulty was observed in single patients and closer to the end of treatment.
A2	Kelly et al., 2018; USA	Descriptive cross-sectional study	To describe discussions of costs of oncology treatment between patients and oncologists in real time and analyze whether these discussions caused discomfort to patients or not.	96 patients with previously treated breast, lung or metastatic colorectal cancer.	28% of oncologists felt comfortable discussing costs; 6% asked patients regularly about financial difficulties; 80% of patients wanted to receive information about costs and 84% reported that such conversations would be important; 72% of patients answered that no healthcare professional discussed the costs with them.
A3	Souza et al., 2017; USA	Methodological research	To correlate patient-reported financial toxicity with health-related quality of life (HRQOL) and investigate whether the Comprehensive Score for financial Toxicity (COST) measure was related to its psychometric properties.	375 patients with stage IV cancer	Correlation with HRQOL indicated that financial toxicity was a clinically relevant, patient-centered outcome.
A4	Zafar et al., 2013; USA	Exploratory and observational study	To report the experiences of insured cancer patients seeking co-payment assistance and analyze the impact of health conditions on well-being and treatment.	254 patients with solid tumors receiving chemotherapy or hormone therapy.	75% of patients applied for drug payment assistance; 68% reported reduced paid activities, 46% reduced spending on basic food and clothing, 46% used their savings, and 17% sold goods or properties to pay for treatment costs. To save money, 20% bought less than the total of prescribed medication; 19% bought part of the prescribed medication; 7% of patients avoided procedures; 9% avoided exams; 4% ignored ways to save money.
A5	Honda et al., 2018; Japan	Methodological research	To analyze the viability of using the Japanese version of the COST questionnaire in the measurement of financial toxicity among Japanese cancer patients.	11 cancer patients undergoing chemotherapy.	Five (45%) patients had degree 1 of financial toxicity and two (18%) patients had degree 2. The COST measure showed good internal consistency with Cronbach’s α of 0.87.

## Discussion

The absence of the term ‘financial toxicity’ in articles prior to 2013 reveals that this concept is new and little used in the literature. Although the studies included in this review aimed to verify, describe or evaluate financial toxicity among cancer patients, the authors used other terms to describe their objectives and raised questions related to the cost of treatment and its relationship and/or impact on well-being and/or QOL.

The term financial toxicity was first used in 2009 to describe the financial impact of cancer treatment (Connor et al., 2016). From this period on, it was used by other authors such as Zafar et al., (2013), Delgado-Guay et al., (2015), and Souza et al., (2014). In 2014, the Functional Assessment of Chronic Illness Therapy (FACIT) group developed an instrument to measure the financial toxicity of patients undergoing cancer treatment called the Comprehensive Score for financial Toxicity (COST-FACIT).

In July 2018, the COST - FACIT was available in the English and Brazilian Portuguese languages. However, by August 2019, it was available in nine languages, showing that the scale is being studied in several countries, as observed in A3 and A5. It is worth mentioning that A3 was the study of validation of the COST, which took place three years after its creation.

The financial problems of patients undergoing cancer treatment have been focus of attention since 1993, when the QLQ-C30 tool was used to measure the overall QOL of patients undergoing cancer treatment. This tool contains an item that assesses the financial problems caused by the physical condition or medical treatment, showing that there has been a concern with this relationship for over 20 years.

Financial toxicity has been linked to several clinically relevant outcomes including QOL, symptom burden, acceptance of the disease, and, recently, survival and treatment adherence (Pollack, 2009; Souza et al., 2014). In the population-based study by Fenn et al. (2014) in which the relationship between financial problems and self-reported QOL was characterized, it was found that the 2,108 patients who reported that cancer caused “many” financial problems were four times less likely to classify their QOL as “excellent”, “very good” or “good”.

Financial toxicity is closely associated with QOL because patients with financial problems may experience difficult situations, such as choosing which bills to pay in the attempt to defray treatment costs, implying the possibility of personal and family bankruptcy (Ramsey et al., 2013) and the onset of feelings of depression and anxiety that negatively impact QOL (Fenn et al., 2014).

For the authors Connor et al. (2016) and, Zafar and Aberneth et al., (2015), there are two types of financial burdens caused by cancer treatment: the objective burden, consisting of the extra expenses with medication, outpatient care and hospitalizations, and the subjective burden that is related to possible changes in well-being and care quality, which are key components of financial toxicity. Thus, extra expenses related to cancer treatment are similar to physical toxicity, as costs may decrease QOL and prevent adherence to care guidelines and proposed therapy (Zafar et al., 2013; Zafar et al., 2015; Neugut et al., 2011; Streeter et al., 2011).

Non-adherence to the indicated therapy contributes to savings, but also increases the risk of adverse consequences. The study by Osterberg and Blaschke conducted in 2005 showed that 33% to 69% of all US drug-related hospitalizations were attributed to non-adherence to treatment, resulting in an annual cost of up to US $ 100 billion. That is, in the short or long-term, the patients’ QOL is compromised because the treatment is not adequate and/or duly completed. Non-adherence to treatment causes side effects that could be managed, culminating in the worsening of the condition and leading to unnecessary hospitalizations or even death. 

In the study by Zafar et al., (2015) with cancer survivors, the authors observed that financial burden was associated with worse QOL. In this sense, the study by Delgado-Guay et al., (2015) that examined the frequency of financial difficulty in terminal cancer patients found that financial discomfort among patients was significantly associated with anxiety, depression and QOL. These studies expose the suffering of patients undergoing cancer treatment who often find themselves powerless before their condition, because their treatment generates unexpected expenses and at the same time they are unable to help with the family budget.

Discontinuity or abandonment of cancer treatment was present in the study A4 and has been widely studied as a consequence of financial toxicity (Zafar et al., 2013; Streeter et al., 2011). In the research by Bestvina et al. (2014) with 300 cancer patients, 27% of the patients reported non-adherence to medication due to financial concerns. Another study by Zafar et al., (2013) showed that an increasing proportion of cancer patients are at risk of cutting back or reducing expenses with groceries, selling their homes, and not adhering to the prescribed treatment.

The abandonment, substitution or reduction of medications prescribed in conventional therapy need to be addressed in dialogues with the health team so that the patients may expose their doubts, difficulties and collaborate in decision-making when it comes to their care, thus reducing their suffering regarding costs and anxieties about the course of treatment or disease. For this, health teams need to provide patients with a moment of conversation in order to allow them to talk about their needs.

In a study by Hamel et al., (2017) conducted at two hospitals in Detroit (USA) to assess the extent and nature of discussions about cancer treatment costs between a sample of African/American patients and their oncologists, it was observed that conversations about costs happened at 45% of clinical interactions and patients started 63% of the discussions. The study also showed that the most frequent concerns of patients were related to work leave for treatment, followed by concerns with insurance, transportation costs, job loss, current treatment expenses, and general financial concerns.

This review points to a lack of studies on this subject, possibly due to the fact that the term financial toxicity is recent. It was observed that financial toxicity causes impairments in QOL, maintenance of treatment, and may boost the use of unconventional therapies. The study points to the importance of investigating financial toxicity because the forecast of costs can help patients to make important decisions about treatment as early as in the moment of diagnosis.

## Statement conflict of interest

We declare for publishing purposes that this article is not being evaluated by any other journal and it has never been published before.
